# Complete mimicry: a case of alveolar rhabdomyosarcoma masquerading as acute leukemia

**DOI:** 10.1186/s13000-017-0667-7

**Published:** 2017-11-02

**Authors:** Osamu Imataki, Makiko Uemura, Shumpei Uchida, Shigeyuki Yokokura, Akihiro Takeuchi, Ryo Ishikawa, Akihiro Kondo, Kayoko Seo, Norimitsu Kadowaki

**Affiliations:** 1 Division of Hematology, Faculty of Medicine, Kagawa University, Kagawa, Japan; 2 Division of Laboratory Medicine, Faculty of Medicine, Kagawa University, Kagawa, Japan; 3 Department of diagnostic pathology, Kagawa University, Kagawa, Japan; 4 Division of Hematology, Department of Internal Medicine, Faculty of Medicine, Kagawa University, 1750-1 Ikenobe, Miki-cho, Kita-gun, Kagawa, 761-0793 Japan

**Keywords:** Rhabdomyosarcoma, Acute leukemia, Mimicking, Flow cytometry

## Abstract

**Background:**

A small number of rhabdomyosarcoma (RMS) cases involve the bone marrow. A leukemic presentation of RMS has been reported in a few case series, although almost all cases of leukemic RMS are not completely mimicking leukemia. We encountered a case with RMS cell infiltration of the bone marrow that resembled floating hematological cells.

**Case presentation:**

We encountered a rare case of a 15-year-old boy with a 2-week history of left femoral pain. Upon admission, he was afebrile with no other symptoms. No apparent cause of femoral pain was detected on an initial examination. Laboratory findings revealed normal white blood cell (WBC) count and hemoglobin concentration, with a platelet count of 10.3 × 10^4^/μL. WBCs included 2.0% metamyelocytes, 4.5% myelocytes, and 0.5% blasts. Lactate dehydrogenase concentration was 1299 U/L, creatine kinase was 437 U/L, and C-reactive protein was 1.25 mg/dL. Bone marrow aspiration demonstrated hypercellular marrow (nucleated cell count 1.84 × 10^4^/μL) and 89.0% of blast-like cells of all nucleated cells. The proliferating cells were negative for myeloperoxidase and esterase, and strongly positive for CD56. Positron emission tomography exhibited extensive accumulation of ^18^F–fludeoxyglucose with a SUVmax of 7.09. Magnetic resonance imaging revealed T1-low intensity, gadolinium-enhanced, diffuse, and irregular lesions on his pelvis and bilateral femurs. These laboratory and imaging findings suggested hematological malignancy with diffuse bone involvement, suggestive of acute leukemia. However, the pathological diagnosis of bone marrow and basal penile muscle biopsy was alveolar RMS. Karyotype analysis of bone marrow cells revealed the characteristic translocation of t(2;13)(q35;q14). The final diagnosis was alveolar RMS with massive involvement of the bone marrow and the primary site in the perineal muscles. The tumor cells both of the primary site and bone marrow were positive for myogenin.

**Conclusions:**

A literature review found a misdiagnosed case of completely mimicking leukemic RMS as natural-killer (NK)-cell leukemia. Such a misdiagnosis can have critical consequences. We experienced a rare case of alveolar RMS with symmetrical diffuse bone marrow involvement completely masquerading as acute leukemia. The results of a surface marker study showing that the tumor cells had a near NK-cell phenotype were misleading.

## Background

Approximately 6–23% of rhabdomyosarcoma (RMS) cases involve the bone marrow [1, 2]. A leukemic presentation of RMS has been reported in a few anecdotal case reports [2–9]. Massive bone marrow involvement of RMS is not unusual, but mimicking of leukemia is rare instance. Especially, diffuse bone marrow involvement of RMS with unknown primary site might be diagnosed as leukemia [7]. In almost all cases of leukemic RMS, an unusual manifestation, such as leukemia, has been elucidated by atypical clinical characteristics, such as formation of a primary lesion, through the course of diagnosis. However, the most relevant information to discriminate complete mimicry of leukemic RMS without an apparent primary mass with leukemia remains unclear.

## Case presentation

**Fig. 1:**
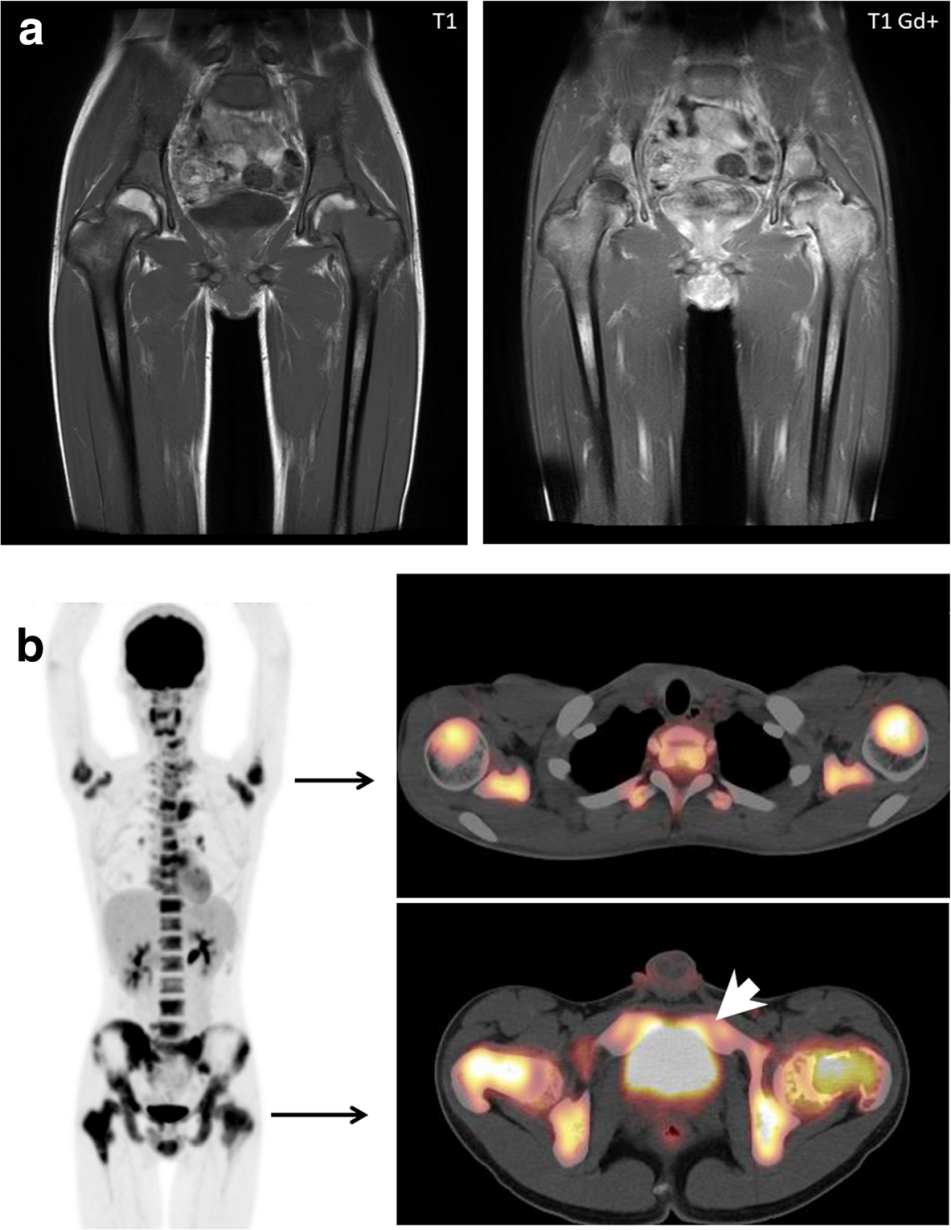
Clinical imaging. **a** Magnetic resonance imaging. Tumors replaced massive amounts of tissue at the bilateral femoral heads, which showed low intensity on T1-weighted images (left) with gadolinium enhancement (right). **b** ^18^F–FDG-PET/CT. Uptake of ^18^F–FDG was symmetrical in the bilateral upper arms and femoral heads, and in the pelvis. Several areas of spotty uptake were also observed in the vertebrae. A transverse image at the level of the upper arm head (right top). A transverse image at the level of the femoral head (right bottom). The white arrow head indicates uptake of perineal muscle, which was recognized as a primary site. (Abbreviations: ^18^F–FDG, 1^8^F–fludeoxyglucose; PET, positron emission tomography; CT, computed tomography)

A 15-year-old boy who initially visited the outpatient orthopedic clinic because of a 2-week history of left femoral pain was admitted to the Division of Orthopedics. On admission, he was afebrile and complained of no other symptoms. An initial examination failed to determine the cause of femoral pain. Magnetic resonance imaging of the pelvis and bilateral femurs on admission revealed T1-weighted, gadolinium-enhanced, low-intensity, diffuse, and irregular lesions on his pelvis and bilateral femurs (Fig. 1a). This imaging indicated a hematological malignancy with diffuse bone involvement, suggestive of acute leukemia. Positron emission tomography (PET) exhibited extensive accumulation of ^18^F–fludeoxyglucose (FDG) with a maximum standardized uptake value of 7.09 (Fig. 1b). The patient’s blood count was normal, although the platelet count was slightly decreased to 10.3 × 10^4^/μL. Some immature myeloid cells (2.0% metamyelocytes, 4.5%, myelocytes, and 0.5% blastocysts) were circulating in the peripheral blood. Laboratory findings showed elevated lactate dehydrogenase (LDH) of 1299 U/L, creatine kinase (CK) of 437 U/L, and C-reactive protein of 1.25 mg/dL. The tumor markers sIL-2R and *WT1* mRNAs were elevated to 566 U/mL (standard range: 135–483 U/mL) and 110-copies/μg RNA (standard range: <50 copies), respectively. These laboratory and imaging findings suggested hematological malignancy. Therefore, the primary orthopedic surgeon consulted a hematologist and a pediatrician, who suggested an immediate bone marrow examination.

**Fig. 2:**
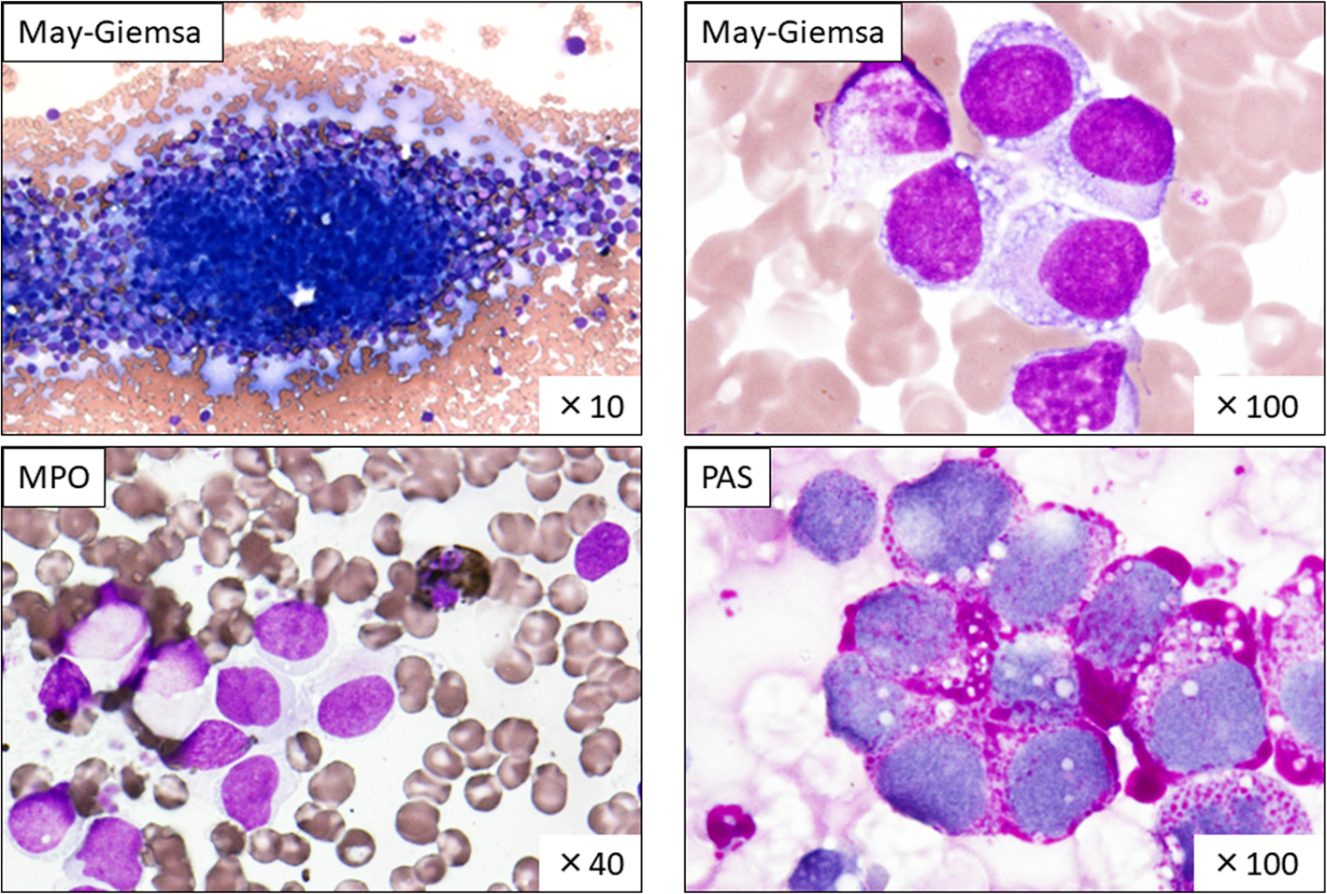
Bone marrow aspiration cytology The cell count was 1.84 × 10^4^/μL and the megakaryocyte content was <6 cells/μL. The M/E ratio was 7.80/4.88. Myeloblasts accounted for 0.0% and blast-like abnormal cells for 89.0%. May–Giemsa staining indicated a diffuse proliferation of basophilic blastic cells, which harbored gross vacuoles in the cytoplasm. The blastic cells were negative for myeloperoxidase  (MPO) and positive for periodic acid–Schiff (PAS) staining

**Fig. 3:**
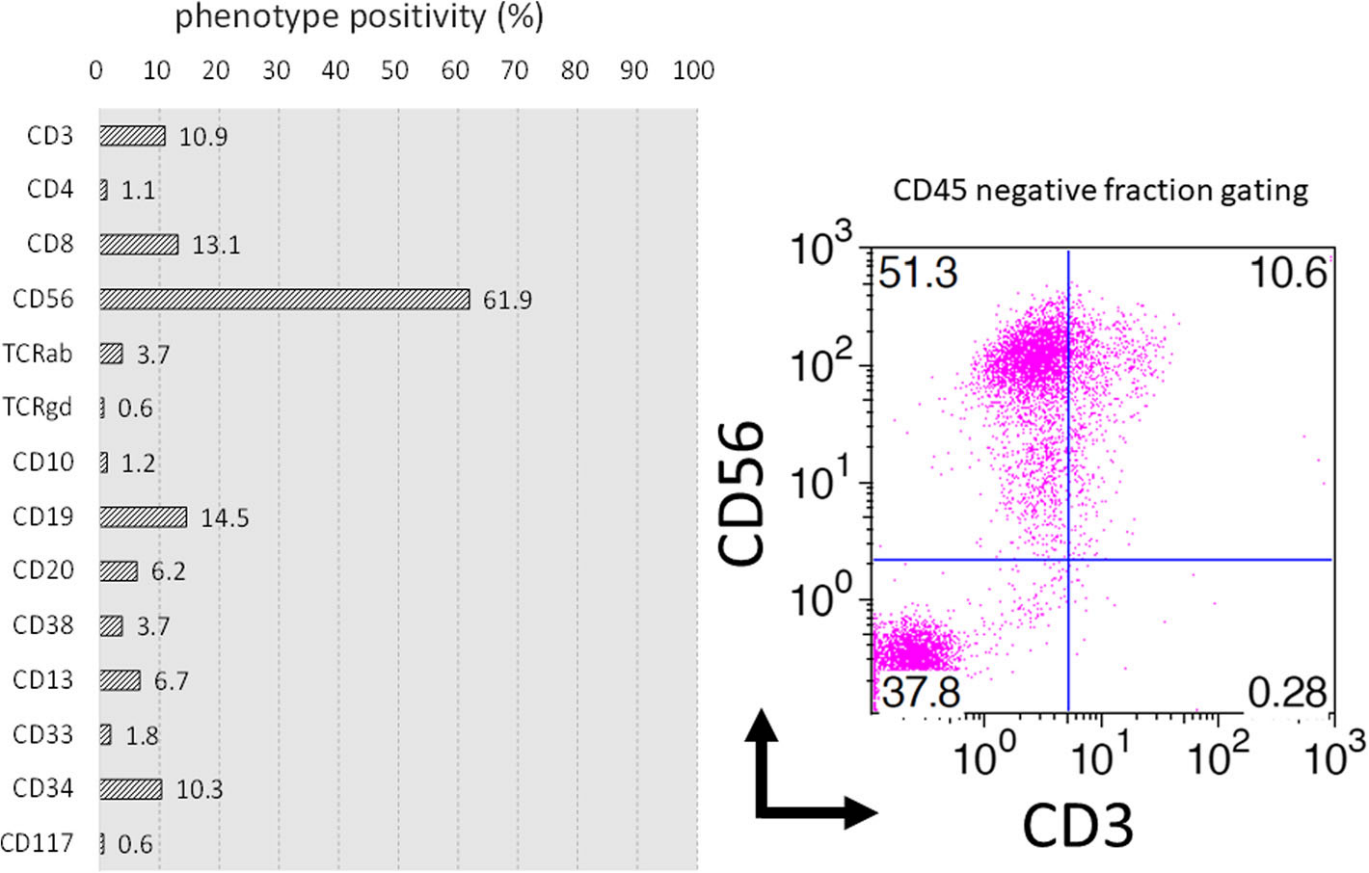
Flow cytometry of bone marrow Flow cytometry analysis indicated that the infiltrating tumor cells were phenotypically positive only for CD56. The indicated proportions of cells in each phenotype were the percentages of the CD45-negative fraction

Bone marrow aspiration (nucleated cell count, 1.84 × 10^4^/μL) demonstrated hypercellular marrow with proliferation of blast-like bullous atypical cells, accounting for 89.0% of all nucleated cells (Fig. 2). The use of special stains for aspirated bone marrow cells showed that the proliferating cells were negative for myeloperoxidase and esterase, but strongly positive for periodic acid–Schiff (Fig. 2). Flow cytometry analysis indicated that the cells were phenotypically positive for CD56 only (Fig. 3). A bone marrow biopsy was simultaneously performed, but the results were delayed because of decalcification. Meanwhile, CK levels were elevated and disseminated intravascular coagulation developed; thus, intravenous hydration therapy was initiated to protect renal function. However, no specific therapy was started while waiting for the confirmation of pathological diagnosis.

**Fig. 4:**
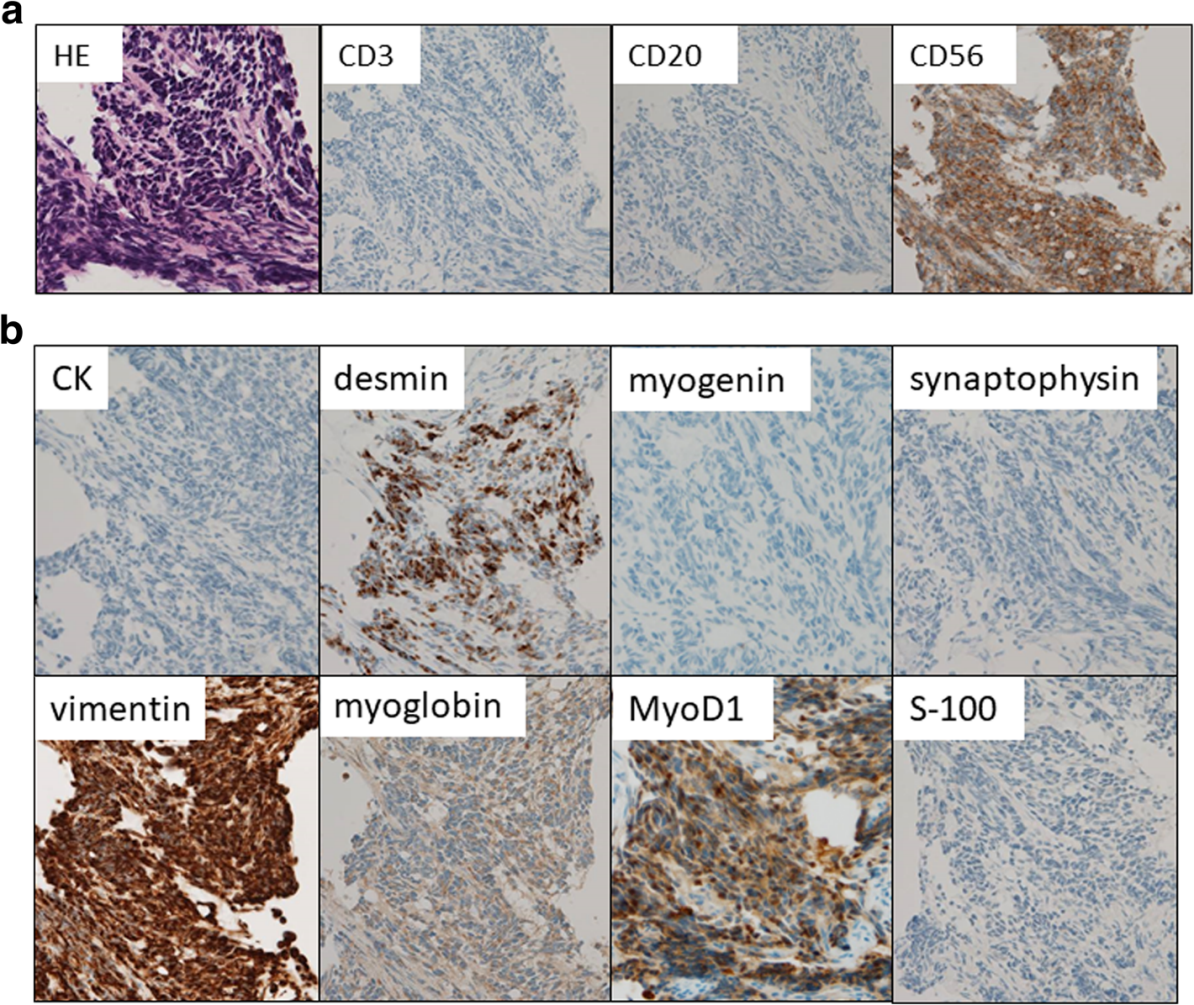
Pathology of tumor in bone marrow. **a** Biopsied bone marrow contained small round tumors with dense nuclei diffusely aggregated and focally floating within fibrous nests. Immunohistochemical staining for specific hematological lineages were negative for CD3 and CD20, but positive for CD56. **b** An additional immunohistochemical study suggested positivity for vimentin. Staining for the muscular differentiation markers desmin, myoglobin, and myogenin were positive, whereas CK and MyoD1 were negative. Staining for the neural differentiation markers synaptophysin and S-100 was negative. Original magnification of 40 ×

**Fig. 5:**
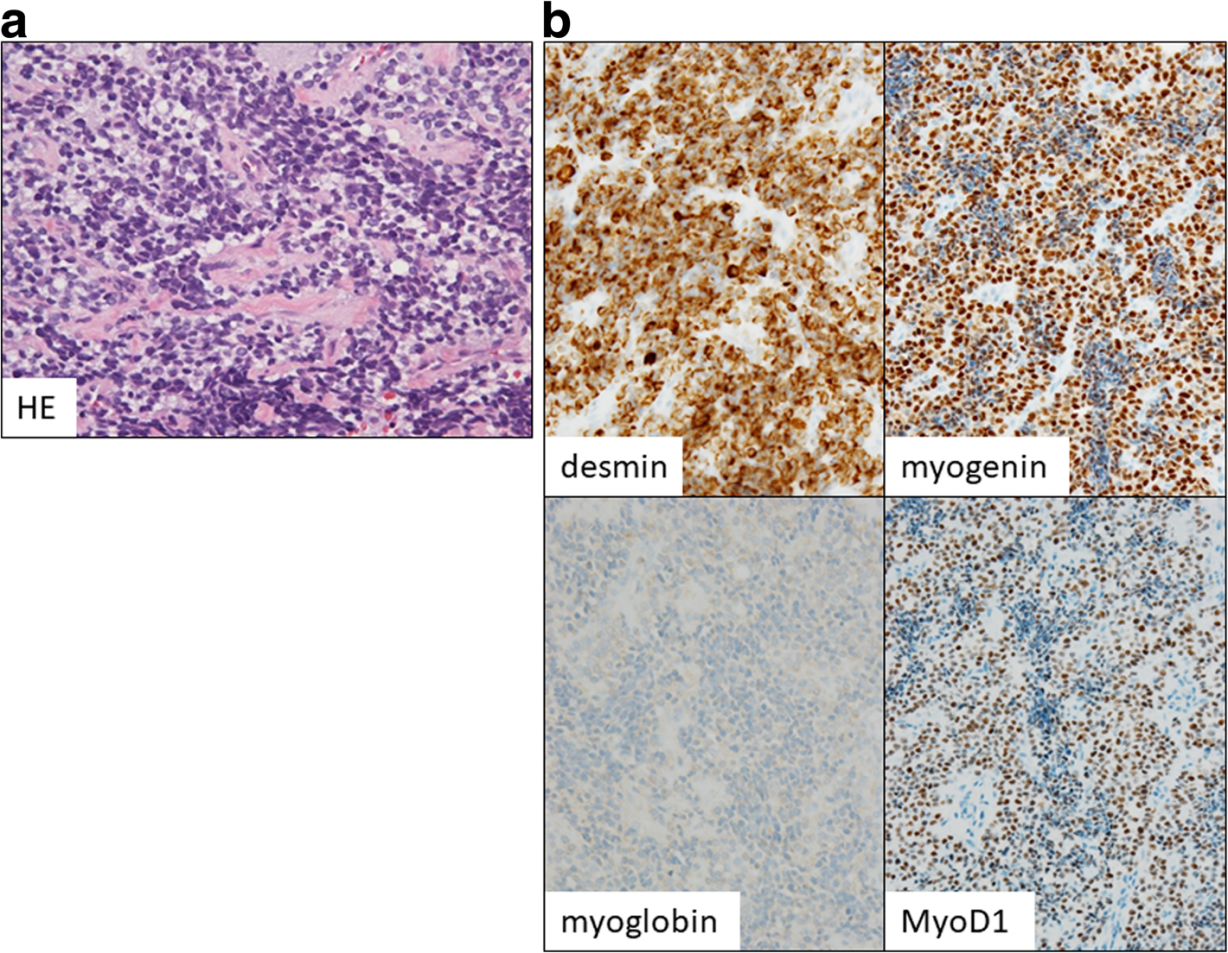
Pathology of tumor in perineal muscle. **a** Rebiopsy of perineal muscle indicated similar pathological features characterized as alveolar RMS and confirmed the pathological diagnosis. **b** An immunohistochemical staining revealed positivity for vimentin, desmin, myoglobin, myogenin and MyoD1 were all positive, whereas CK, synaptophysin, and S-100 were negative. Original magnification of 40×

**Fig. 6:**
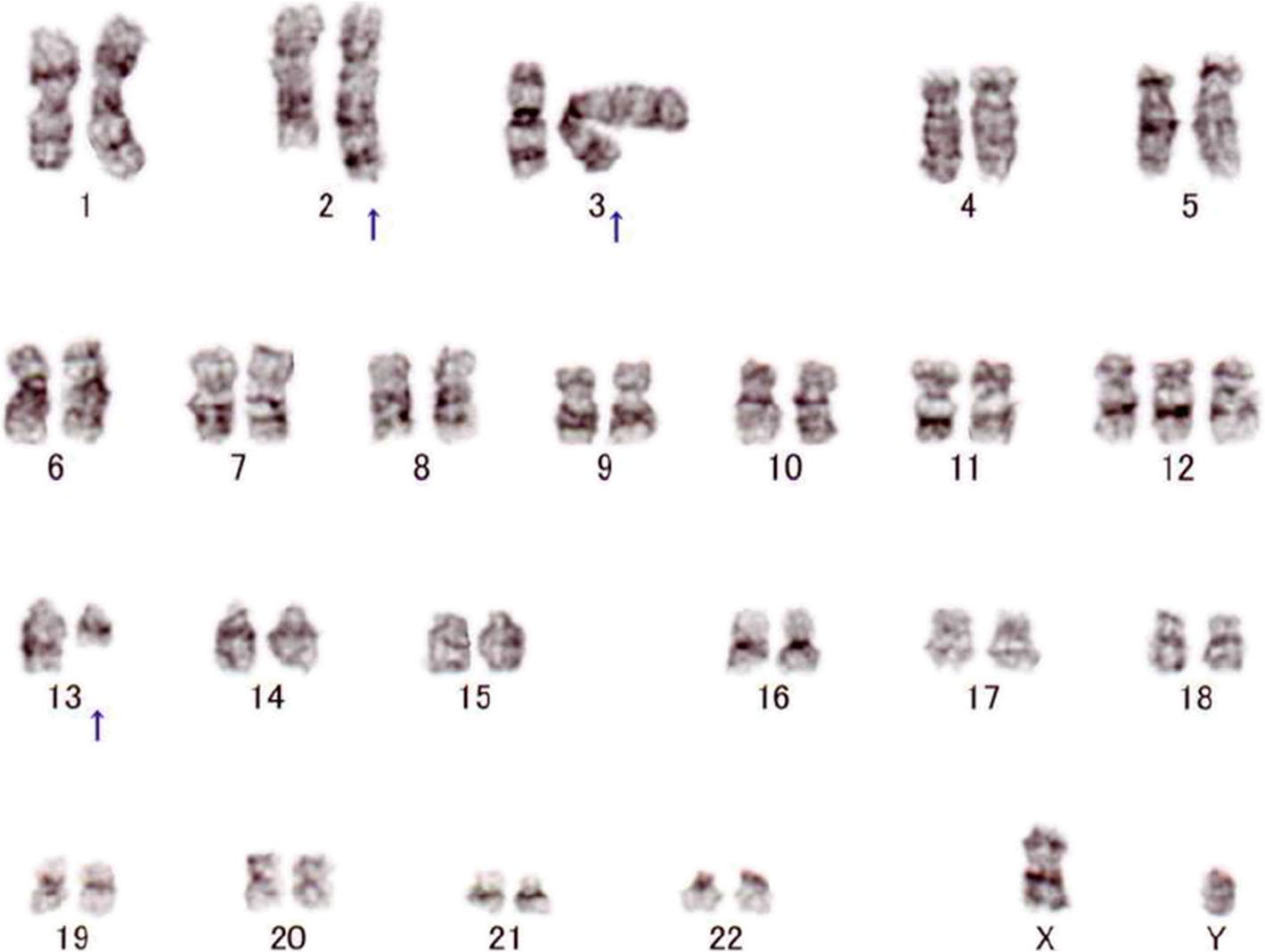
Chromosomal analysis of bone marrow cells. A karyotype of bone marrow cells revealed translocation of (2;13)(q35;q14) in 14 among 20 analyzed cells

After a delay of a couple of weeks, supportive hydration therapy was initiated and the pathological examination of the bone marrow biopsy based on the curious morphology and positivity for CD56 (Fig. 4a), desmin, vimentin, myoglobin, and myogenin (Fig. 4b) confirmed a tentative diagnosis of alveolar RMS. The tumor cells were negative for synaptophysin, S-100, and MyoD1 (Fig. 4b). Medical oncologists and radiologists reviewed the ^18^F–FDG-PET images and recognized a primary mass in the penile region, which was initially overlooked. Basal penile biopsy was performed by an urologist and the results of a histological examination of the biopsied tissue were in agreement with the pathological findings of the bone marrow (Fig. 5a). The immunohistochemical staining pattern was similar to that of the bone marrow sample: i.e., positive for desmin, vimentin, and myoglobin, and negative for synaptophysin, S-100. However, of note, myogenin and MyoD1 staining were both positive (Fig. 5b). The histopathology of basal penile muscle indicated characteristic alveolar growth of small round cells with round regular nuclei and scant cytoplasm, which led to a diagnosis of alveolar RMS. Afterwards, karyotype analysis of bone marrow cells revealed t(2;13)(q35;q14) (Fig. 6), which is a characteristic translocation of RMS, producing the PAX3-FOXO4 (FKHR) fusion protein. The final diagnosis of this patient was alveolar RMS with massive involvement of the bone marrow and the primary site in the perineal muscles (Fig. 1b, arrow head). The patient was transferred to a hospital that specializes in pediatric oncology. The Japanese sarcoma study group protocol was started with an initial treatment regimen for high risk patients, consisting of vincristine, doxorubicin, and cyclophosphamide (VDC) followed by ifosfamide and etoposide (IE), which was designating to coordinate with future stem cell transplantation. He responded well to this induction treatment and received radiation therapy for the residual disease.

## Discussion

Among several reported cases of leukemic RMS, the present case had completely mimicked leukemia [1–9]. A search of the literature revealed that leukemic RMS cases were first detected several decades ago by specialists in a variety of medical fields, including pediatrics, hematology, pathology, and pediatric surgery [1–3, 7, 8]. Possible reasons for the wide spectrum of specialties concerned with this disease include the following: (1) RMS occurs in adolescents and young adults, (2) RMS can initially involve many organs with striated muscle, including the head and neck, extremities, and genitourinary system, and (3) symptoms at the onset varies among cases as fever, pain, anemia, or other unspecific symptoms. These clinical manifestations can delay consultation with a physician. Intervention by a multi-disciplinary oncological team and the integration of trans-division consultation is effective in achieving an accurate diagnosis.

In adult onset RMS, the genitourinary system is the most common site of a primary lesion [3, 5, 7], while other primary sites may remain unknown [2–4, 6, 7, 9]. In the present case, the penile muscle was considered as the primary site of RMS. An extensive search for a primary site of RMS in the current case using PET/computed tomography (CT) showed perineal accumulation of ^18^F–FDG with diffuse symmetrical bone marrow accumulation. In this case, ^18^F–FDG-PET/CT was quite useful not only to detect the primary lesion but also to differentiate RMS from acute leukemia. However, in sarcoma cases with unknown primary sites, RMS mimicking leukemia can lead to misdiagnosis and inadequate treatment [7]. The presence of atypical blasts lacking CD45, a common hematopoietic marker, suggests an alternative to suspected sarcoma, such as RMS with an unknown primary site. In such cases, bone marrow biopsy is an effective method to confirm diagnosis.

This patient required intensive treatment to achieve cure of disease. He was transferred to a community hospital that specialized in pediatric oncology and received the Japanese sarcoma study group protocol. Fortunately, the initial treatment regimen (VDC–IE) was effective. His disease was sufficiently sensitive to achieve partial response. Surprisingly, an unfortunate case misdiagnosed as natural killer (NK) cell leukemia was reported previously [7]. That case was treated for primary refractory NK-cell leukemia with a protocol designed for treatment of acute leukemia before undergoing stem cell transplantation. This case was finally diagnosed as RMS based on the recurrence of disease after transplantation. A misdiagnosis at the initial onset of disease can have critical consequences. In our case, intervention by a multi-disciplinary oncological team and the integration of trans-division consultation was effective to arrive at an accurate diagnosis.

## Conclusion

We experienced a rare case of alveolar RMS with symmetrical diffuse bone marrow involvement completely masquerading as acute leukemia. The results of a surface marker study showing that the tumor cells had a near NK-cell phenotype were misleading.

## Abbreviations

CK: Creatine kinase
CT: Computed tomography
FDG: ^18^F–fludeoxyglucose
LDH: Lactate dehydrogenase
NK: Natural killer
PET: Positron emission tomography
RMS: Rhabdomyosarcoma
